# Peritonitis from Anaerobic Gram-positive Cocci Likely Due to Translocation of Bacteria from Gut in a Patient Undergoing Peritoneal Dialysis

**DOI:** 10.7759/cureus.6060

**Published:** 2019-11-03

**Authors:** Sreedhar Adapa, Srikanth Naramala, Daniel Boken, Alan Moreno, Venu Madhav Konala

**Affiliations:** 1 Internal Medicine/Nephrology, Kaweah Delta Medical Center, Visalia, USA; 2 Rheumatology, Adventist Medical Center, Hanford, USA; 3 Internal Medicine/Infectious Disease, Kaweah Delta Medical Center, Visalia, USA; 4 Internal Medicine/Hematology and Oncology, Ashland Bellefonte Cancer Center, Ashland, USA

**Keywords:** enterococcus avium, peritonitis, enterococcus

## Abstract

Peritonitis is a severe complication encountered in patients undergoing peritoneal dialysis, often causing high morbidity and mortality. High vigilance is required from healthcare providers involved in the management of these patients to prevent this complication. There has been recognition of peritonitis caused by unusual organisms because of improved microbiological detection techniques. Gram-positive organisms are the frequent cause of peritonitis compared to Gram-negative organisms. We report a rare case of peritonitis caused by Enterococcus avium. A 60-year-old male on peritoneal dialysis presented with palpitations, abdominal pain, diarrhea, and cloudy effluent. The peritoneal fluid analysis was consistent with peritonitis, and peritoneal fluid culture grew E. avium. The patient was treated with linezolid after failing to respond to vancomycin. The peritoneal dialysis catheter was removed, and the dialysis modality has been switched to hemodialysis because of refractory peritonitis.

## Introduction

*Enterococcus avium* is Gram-positive, catalase-negative cocci, and facultative anaerobe [1]. It is a normal commensal of the gastrointestinal and genitourinary tract in humans and animals. The organism was isolated initially from human feces in 1955 [2]. Peritonitis is a significant complication encountered in patients undergoing peritoneal dialysis, which causes significant morbidity and mortality [3]. The incidence of peritonitis from rare organisms is increasing because of improved microbiological identification techniques. We report a case of *E. avium* associated peritonitis.

## Case presentation

A 60-year-old male was admitted with a chief complaint of palpitations, abdominal pain, and diarrhea. Palpitations were sudden in onset and woke up the patient from sleep and were persistent, which prompted him to seek medical attention. The patient was having diarrhea and abdominal pain for two days before the presentation. Past medical history was significant for polycystic kidney disease resulting in end-stage renal disease, bilateral nephrectomies with living unrelated kidney transplant that failed after 13 years, and he was on peritoneal dialysis for two years. Other history details include hypertension, diabetes, hyperlipidemia, and coronary artery disease with four-vessel coronary artery bypass grafting. Home medications include aspirin 81 milligrams (mg) daily, ticagrelor 90 mg twice a day, calcitriol 0.25 micrograms daily, carvedilol 6.25 mg twice a day, olmesartan 40 mg daily, vitamin d2 50,000 units weekly, and insulin sliding scale. The vital signs in the ER were a temperature of 36.5 centigrade, pulse rate of 80 beats per minute (bpm), respiratory rate of 18 breaths per minute, and blood pressure of 123/76 mmHg. Physical examination revealed that the patient was in sinus rhythm, with no rubs or gallops or murmurs. Abdominal examination revealed a distended, tender abdomen with a peritoneal dialysis catheter in the left lower quadrant. The rest of the physical examination was unremarkable.

**Figure 1:**
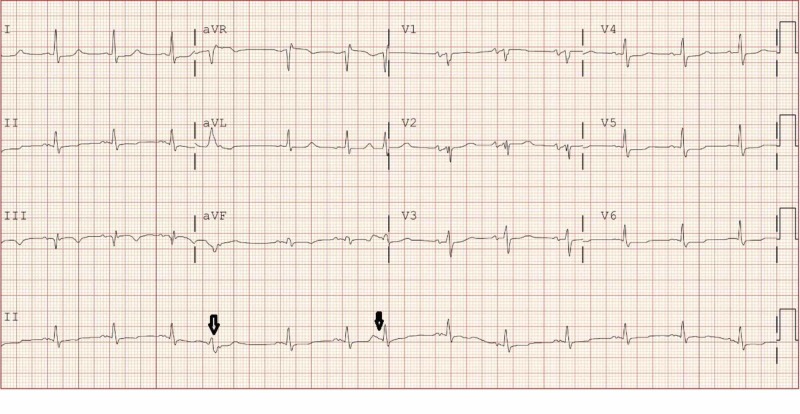
Electrocardiogram - sinus rhythm at 80 beats per minute and premature complexes shown in black arrows.

Investigations revealed electrocardiogram with 80 bpm in sinus rhythm, premature complexes, and nonspecific conduction delay as shown in Figure 1. Laboratory data showed hemoglobin 7.8 g/dL, white blood cell (WBC) count 7220 mm^3^, platelet count 150,000 mm^3^, sodium 131 mmol/L, potassium 4.3 mmol/L, bicarbonate 20 mmol/L, blood urea nitrogen 76 mg/dL, creatinine 12.92 mg/dL, albumin 2.8 g/dL, and troponin I 15.01 ng/mL. The peritoneal fluid effluent revealed peritoneal fluid WBC 14,309 cells/uL, with 89% predominant neutrophils. Peritoneal fluid Gram stain revealed >100 WBC, and no organisms were seen. The patient was started on treatment for peritonitis with empiric intraperitoneal vancomycin and ceftazidime. The patient underwent coronary intervention because of non-ST elevation myocardial infarction (NSTEMI) and had successful angioplasty of in-stent restenosis of ramus intermedius. The peritoneal fluid WBC count started to get better with 11,715 cells/uL on day 2,4502 cells/uL on day 3,1574 cells/uL on day four and got worse to 10,097 cells/uL on day 5. Peritoneal fluid culture grew *E. avium* in both aerobic and anaerobic bottles. Species identification was made by VITEK 2. Sensitivities were done using a broth microdilution technique. The sensitivities of *E. avium* to antibiotic are listed in Table 1.

**Table 1:** Antibiotic sensitivities of Enterococcus avium. S - sensitive

Antibiotic	MIC (minimum inhibitory concentration)	Antibiotic sensitivity
Ampicillin	<=4 mcg/mL	S
Daptomycin	0.5 mcg/mL	S
Linezolid	1 mcg/mL	S
Vancomycin	<=0.5 mcg/mL	S

The patient was initially treated with vancomycin and was changed to linezolid because of worsening peritoneal fluid cell count before the antibiotic sensitivities were available. The peritoneal dialysis catheter was removed, and the dialysis modality has been switched to hemodialysis because of refractory peritonitis. The patient was discharged on linezolid and completed the antibiotic course for a total of 14 days. He was followed up in the clinic after completion of antibiotics and was doing well without any signs and symptoms of recurrent infection. He was continued on hemodialysis three times a week as an outpatient for end-stage renal disease.

## Discussion

Enterococcus species are normal commensals of human gut flora. They cause urinary tract infections, intra-abdominal infections, bacteremia, and nosocomial infections that are antibiotic-resistant frequently [4]. Ninety percent of infections caused by Enterococcus species are attributed to *E. faecalis* and *E. faecium* [5]. Other clinically important species in Enterococci are *E. avium*, *E. flavescens*, *E. casseliflavus*, and *E. gallinarium*, which accounts for less than five percent of clinically significant infections [5].

*Enterococcus avium* was formerly placed in group Q streptococcus as it has group Q antigen [6]. *Streptococcus avium* derives its name as they were frequently isolated from chicken feces [6] and subsequently were isolated from feces of all mammals. The bacterium was later transferred to genus Enterococcus in 1980 as the biochemical features were similar to Enterococcus species [7].

*Enterococcus avium* has low virulence [8] and is an opportunistic pathogen in an immunocompromised host. The bacterium colonizes in the gastrointestinal and genitourinary tract, and the mode of transmission could be from contamination of devices or translocation from the colonized body sites. Most of the case reports or series that had been reported in the literature have severe gastrointestinal diseases. They are often associated with other gastrointestinal organisms, and infections tend to be polymicrobial [9]. *E. avium* has been isolated from the blood and bile of immunocompetent host with acute cholecystitis before [10]. *E. avium* has been reported to cause bacteremia, meningitis, endocarditis, and intra-abdominal infections. *E. avium* rarely causes peritonitis, but only a few cases were reported so far as per the review of the literature [11-12].

*Enterococcus avium* is readily identified by the routine blood and body fluid culture. Commonly used media to identify Enterococcus species include bile esculin agar and 6.5% salt broth [12]. Species identification techniques include VITEK 2 automated system, matrix-assisted laser desorption/ionization-time of flight (MALDI-TOF), polymerase chain reaction (PCR) for specific genes, 16S rRNA sequencing, and proprietary multiplexed nucleic acid amplification [13-16].

*Enterococcus avium* is susceptible to most of the antibiotics, which differentiates, from *E. faecalis* and *E. faecium* that tend to be resistant to them [9-10]. However, our patient was initially treated with vancomycin and responded with decreased peritoneal fluid white cell count and then got worse; hence, vancomycin was switched to linezolid before the sensitivities were available. Prompt identification and timely initiation of antibiotics and appropriate intervention will prevent mortality associated with this organism.

## Conclusions

*Enterococcus avium* is an organism with low virulence which causes opportunistic infections. Peritonitis from *E. avium* in patients receiving peritoneal dialysis is rare and recent diagnostic methods aid in the identification of this organism. The bacterium is usually sensitive to common antibiotics.
